# Differentiation of some Pramenka sheep breeds based on morphometric characteristics

**DOI:** 10.5194/aab-62-393-2019

**Published:** 2019-07-10

**Authors:** Božidarka Marković, Peter Dovč, Milan Marković, Dušica Radonjić, Mirjana Adakalić, Mojca Simčič

**Affiliations:** 1 Biotechnical Faculty, University of Montenegro, 81000 Podgorica, Montenegro; 2 Biotechnical Faculty, University of Ljubljana, 1000 Ljubljana, Slovenia

## Abstract

For the purpose of the morphometric characterization and differentiation of local sheep breeds that belong to the group of breeds called Pramenka or Zackel, two Slovenian (Bela Krajina and Istrian Pramenka) and four Montenegrin sheep breeds (Bardoka, Sjenička, Pivska Pramenka, and Zeta Žuja) were studied. The variation of morphometric measures and nine morphometric indices were analysed. Principal component analysis (PCA) was applied in order to provide an easier description of body size and shape. Regarding body size, the Sjenička breed was one of the largest breeds (body weight 76.4 kg, wither height 72.7 cm, chest circumference 100.3 cm), while Zeta Žuja had the smallest body size (37.1 kg, 64.8, and 81.9 cm). On the other hand, Slovenian Istrian Pramenka had the largest body length, chest depth, chest width, and rump width among all included breeds (79.4, 33.6, 22.7, and 21.2 cm). Bela Krajina, Istrian Pramenka, and the Sjenička breed, according to the index of body frame (IBF) value (107–114), have a rectangular body frame, Bardoka and Pivska Pramenka have a square body frame (99.3–100), and Zeta Žuja has a short body frame (91.8). The PCA of all morphometric parameters extracted three components accounting for 96.6 % of the cumulative variance. An unweighted pair–group method with arithmetic mean (UPGMA) cluster analysis by Euclidian distance shows diversity among the studied breeds, through it grouped Pivska Pramenka with Sjenička and Istrian with Bela Krajina Pramenka in two clusters, while Bardoka and Zeta Žuja were clustered separately.

## 1 Introduction

Genetic diversity among breeds enables the existence of livestock in different environmental conditions and provides a range of products and functions necessary for the human population (Yunusa et al., 2013; Salako and Ngere, 2002). After the domestication of sheep, the main diffusion routes from the Middle and Near East to Europe were through the Balkan region along the Mediterranean and across the Danube valley (Zeder, 2008). In the area of the Balkan Peninsula, many local sheep breeds have evolved under specific geographical and climatic conditions, and most of them belong to the group of coarse wool breeds called Pramenka or Zackel (Porcu and Marković, 2006).

Sheep production in Montenegro is an important livestock sector, with a current population of about 200 000 heads (Marković et al., 2018). There are several local breeds like Pivska Pramenka and Sjenička sheep, which make up the largest share in the total population, while other breeds (Bardoka, Ljaba, Sora, and Zeta Žuja) represent a relatively small part of the population (Adžić et al., 2004; Marković et al., 2007, 2011), but all of them are very valuable from a genome preservation point of view.

The current sheep population in Slovenia is about 120 000 heads (SURS, 2016). Slovenian sheep production is also mostly based on local breeds, including Jezersko–Solčava sheep and Bovška sheep, which belong to the Alpine group of breeds, and the Istrian and Bela Krajina breeds, which belong to the Pramenka group (Kompan and Pogačnik, 1994; Kompan et al., 1996; Žan Lotrič et al., 2013).

Characterization of local domestic animal populations, with a breed as the main operation unit, is very important for livestock diversity assessment (Duchev and Groeneveld, 2006). Breed characterization based on morphological characteristics can provide a reasonable representation of the differences among breeds; it serves as the basis upon which DNA analysis can be built (Yunusa et al., 2013). Morphometric characterization has a large significance in the process of describing phenotypic characteristics and morphological diversity among breeds (Stojiljković et al., 2015). Likewise, the estimated indices from conventional and non-conventional body measurements could serve as indicators of type and function (Salako, 2006). Morphometric indices may provide a relatively easier and more unbiased way to assess body conformation. They are becoming very good indicators of the usefulness of an animal (Khargharia et al., 2015).

Principal component analysis (PCA) is used as an interdependence technique to identify morphometric parameters that best serve as breed-specific markers (Khargharia et al., 2015). Many researchers used PCA analysis to extract factors contributing to variation amongst individual animals based on body measurement (Mavule et al., 2013; Yunusa et al., 2013; Yakubu, 2013; Khargharia et al., 2015). Salako (2006) used PCA for 10 linear body measurements of Uda sheep and reduced them into two principal components that accounted for 75 % of the total variation.

A review of the basic exterior characteristics of the most important local Pramenka sheep breeds in the western Balkan countries was published by Porcu and Marković (2006), while the genetic diversity of seven Pramenka breeds from the western Balkan countries was studied by Ćinkulov et al. (2008). A comparative analysis of morphological and productive traits of local sheep breeds from Montenegro and Croatia was performed by Marković et al. (2013) and Antunović et al. (2015). On the other hand, genetic and phenotype diversity between the Slovenian and Croatian sheep breeds was investigated by Ivanković and Dovč (2004), Šalamon et al. (2015), and Jevšinek Skok et al. (2015). Until now, there has not been any comparative study between Slovenian and Montenegrin Pramenka sheep breeds, despite both countries being on the Mediterranean route for spreading sheep in Europe after the domestication process. The aim of this study was the morphometric characterization and differentiation of four Montenegrin breeds (Pivska, Sjenička, Bardoka, and Zeta Žuja) and two Slovenian breeds (Bela Krajina and Istrian Pramenka).

**Figure 1:**
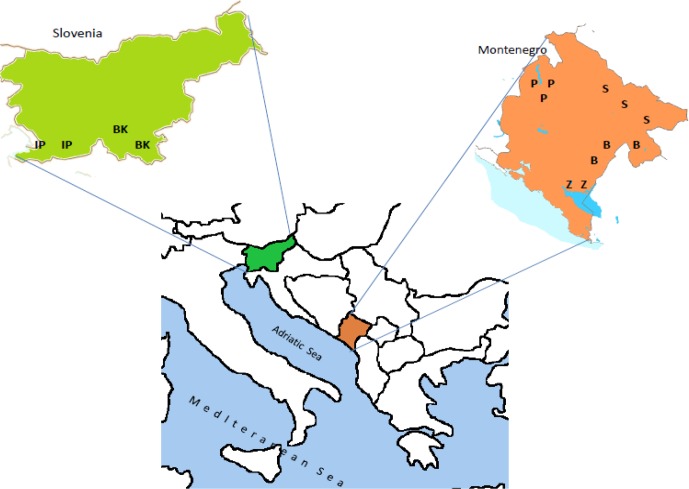
Rearing areas of Slovenian and Montenegrin sheep breeds. (IP – Istrian Pramenka, BK – Bela Krajina Pramenka, P – Pivska Pramenka, S – Sjenička, B – Bardoka, Z – Zeta Žuja).

## 2 Material and methods

Four local Montenegrin sheep breeds and two Slovenian local sheep breeds included in this study are reared in different geographic areas (Fig. 1), under different climate conditions, and in different production systems.

### 2.1 Montenegrin Pramenka sheep breeds

Pivska Pramenka (synonym – Jezeropivska) is a local sheep breed traditionally reared in the north-west part of Montenegro (area in the mountains near Durmitor and Sinjajevina; 1000 to 1800 m a.s.l.). This area is characterized by a mountain climate with long, very cold, and snowy winters. Consequently, Pivska Pramenka is very well adapted to such conditions. This breed has mainly white coarse wool, while 5 %–7 % of the animals are black or gray coloured. The face and forelegs are white with black spots. All males and 50 % of females are horned. The population of Pivska Pramenka is permanently decreasing. The population size is estimated to be about 3000 purebred animals.

The Sjenička sheep is one of the most numerous breeds in Montenegro. The main rearing area is north-east Montenegro (municipalities Berane, Bijelo Polje, and Rozaje) from 600 to 1200 m a.s.l., while crossbreeds are reared throughout Montenegro today. Typical animals have white wool, with black markings around the eyes like eyeglasses, as well as a dark muzzle and ears. All males are horned, while females are polled. This breed has been crossed with more productive breeds in the past, like Merinolandschaf (Württemberg) and some others.

Zeta Žuja is a unique breed easily recognized by the characteristic yellow–brown face and legs, while the wool is white. The rearing area is limited to the flat area around Podgorica (Zeta–Bjelopavlici plain and the basin of Lake Skadar). This area, from 50 to 200 m a.s.l., is characterized by a Mediterranean climate with hot summers and long dry periods. Zeta Žuja has a very small body frame and a low production capacity, while it is resistant and excellently adapted to the very hot climate. The population size of Zeta Žuja is very small, only about 150 heads. It is the most endangered sheep breed in Montenegro with a high risk of extinction.

Bardoka is a sheep breed reared mainly in the south-east of Montenegro along the border with Albania and Kosovo, mostly in areas of the municipality of Podgorica and partly Plav and Gusinje. This breed has long white-coloured fleece with very coarse wool (fibre diameter more than 40 µm); the face and legs are white as well. Ewes are usually polled, while rams are always horned. This breed is characterized by a very good milk yield of up to 200 kg per lactation depending on the grazing quality. The total population size in Montenegro is about 2000 breeding purebred animals.

### 2.2 Slovenian Pramenka sheep breeds

The Bela Krajina Pramenka is a breed widespread in the south-east part of Slovenia used for lamb production. The ewes of Bela Krajina Pramenka weigh over 50 kg, while rams weight 65 to 70 kg. The animals have long coarse wool. The coat colour is mainly white with black spots on the head and legs. The sheep tails are very long, almost reaching the ground. Rams have extremely large horns, which are curled several times according to age. Ewes may have horns as well but shorter (Kompan et al., 1996). Ewes are seasonally fertile with an average of 1.18 lambs per year (Zajc et al., 2015). The population size is 900 animals, and the population is endangered.

The Istrian Pramenka is widespread in the regions of Karst and Istria (Žan Lotrič et al., 2013) and is used for milk production. Rams weigh up to 95 kg, while ewes weigh from 60 to 75 kg. The wool is white, black, or white with black dots and does not cover the whole body. The belly, head, neck, and legs are not covered with wool. Horns are present in rams, while ewes are either horned or polled. The litter size is 1.05 lambs per year (Zajc et al. 2015), and ewes produce 119 kg of milk per lactation. The milk contains on average 7.2 % milk fat and 5.9 % milk proteins. The population size is 1150 animals, and the population is endangered.

### 2.3 Data collection

For the purpose of morphometric characterization, traits of investigated sheep breeds were recorded from flocks reared in their typical areas. A total of 231 animals from Montenegrin sheep breeds (44 Bardoka, 59 Sjenička, 90 Pivska Pramenka, and 38 Zeta Žuja) and 150 animals from Slovenian sheep breeds (86 Bela Krajina Pramenka and 64 Istrian Pramenka) were recorded. All studied animals were randomly selected, and all animals were from 3.0 to 5.5 years old – estimated from dentition for Montenegrin breeds and according to the pedigree data for Slovenian breeds.

Body measurements were taken on animals in a standing position with a raised head. Measurements were done by using a Lydthin stick and flexible measuring tape, while a scale was used for the body weight. The measurements were performed in accordance with the guidelines described by the FAO (2012). Nine morphological traits were measured on each animal: wither height (WH), rump height (RH), body length (BL), chest depth (CD), chest width (CW), rump width (RW), chest circumference (CC), cannon bone circumference (CBC), and body weight (BW). Based on the body linear measurements, respective body size indices were calculated.

The morphometric indices were calculated with the aim of assessing the body proportions as well as the type and functions of the studied sheep breeds. Indices were calculated according to Chacon et al. (2011), Pares-Casanova et al. (2013), Khargharia et al. (2015), and Salako (2006) as follows.

1. *Index of body frame (IBF) or index of length*. This is calculated as body length / wither height × 100. If this measure is larger than 103, the animal has a rectangular body frame or longline; if it is between 97 and 103, animals have a square body frame, while if it is less than 97, the animal is short or brevigline.
2. *Chest index (CI) or thorax index*. This is calculated as chest width / chest depth × 100. It indicates the degree of skeletal fitness of the animal in terms of leg length.
3. *Index of height (IH) or index of body proportion*. This is calculated as wither height / rump height × 100.
4. *Chest depth index (CDI)*. This is calculated as chest depth / wither height × 100.
5. *Index of thorax development (ITD)*. This calculated as chest circumference / wither height × 100. It indicates the thorax development of the animals; animals with relative values above 120 have good thorax development.
6. *Dactyl thorax index (DTI)*. This is calculated as cannon bone circumference / chest circumference × 100; it also indicates thoracic development. DTI is not higher than 10.5 in light animals; it can be up to 10.8 in intermediary animals, up to 11.0 in light meat-type animals, and up to 11.5 in heavy meat-type animals.
7. *Baron–Crevat index (BCI) or index of conformation*. This is calculated as (chest circumference)${}^{2}$ / wither height. The higher the index, the more robust the animal.
8. *Relative cannon bone index (RCBI)*. This is calculated as cannon circumference / wither height × 100.
9. *Index of body weight (IBW)*. This is calculated as body weight / wither height × 100.

The indices CI, IBF, and IH belong to the group of ethnological indices, which contribute to the breed characteristics, whereas the rest (4 to 9) are functional indices, which contribute to information about the type, purpose, and performance of the breed (Esquivelzeta et al., 2011).

### 2.4 Statistical analysis

Descriptive statistics for the morphometric traits were obtained using SAS software (2009). The general linear model (GLM) procedure was used for the assessment of the breed effect on the morphometric traits and indices with the model

1 $$y_{ij}=\mu +B_{i}+e_{ij},$$

where $y_{ij}$ is a trait, μ is the mean, $B_{i}$ is the breed effect (i=1,…,6), and $e_{ij}$ represents random effects.

A Pearson's test was conducted to evaluate correlations between the morphometric traits of investigated sheep breeds.

Principal component analysis (PCA) was performed to identify morphometric parameters that best serve as racial markers for the differentiation of investigated sheep breeds (Mavule et al., 2013; Cerqueira et al., 2011). Analyses were applied for 18 morphometric parameters of all studied breeds. The data were standardized, and a dendrogram plot was constructed using the unweighted pair–group method with arithmetic mean (UPGMA) on the basis of Euclidean distances. These analyses were accomplished using the statistical package Statistica (version 10).

**Table 1:** The least square means (LSMs) with standard errors (SEs) of body measurements (cm) and body weight (kg) for six sheep breeds.

	Sheep breeds					
Type of	Istrian	Bela Krajina	Bardoka	Pivska	Sjenička	Zeta Žuja
trait	Pramenka	Pramenka		Pramenka		
WH	$69.62\pm 0.30^{\text{a}}$	$65.56\pm 0.26^{\text{bc}}$	$66.22\pm 0.63^{\text{c}}$	$71.33\pm 0.25^{\text{d}}$	$72.71\pm 0.31^{\text{e}}$	$64.76\pm 0.39^{\text{b}}$
RH	$68.56\pm 0.29^{\text{a}}$	$65.37\pm 0.25^{\text{b}}$	$66.30\pm 0.35^{\text{c}}$	$71.93\pm 0.25^{\text{d}}$	$73.14\pm 0.30^{\text{e}}$	$65.04\pm 0.38^{\text{b}}$
BL	$79.39\pm 0.35^{\text{a}}$	$72.63\pm 0.30^{\text{b}}$	$66.15\pm 0.42^{\text{c}}$	$70.79\pm 0.30^{\text{d}}$	$77.89\pm 0.37^{\text{e}}$	$59.41\pm 0.46^{\text{f}}$
CD	$33.59\pm 0.21^{\text{a}}$	$32.68\pm 0.18^{\text{b}}$	$29.11\pm 0.25^{\text{b}}$	$32.10\pm 0.18^{\text{d}}$	$33.01\pm 0.22^{\text{bc}}$	$27.26\pm 0.27^{\text{e}}$
CW	$22.69\pm 0.28^{\text{a}}$	$22.42\pm 0.24^{\text{a}}$	$18.83\pm 0.33^{\text{b}}$	$21.79\pm 0.23^{\text{ac}}$	$21.32\pm 0.29^{\text{c}}$	$15.13\pm 0.36^{\text{d}}$
RW	$21.16\pm 0.22^{\text{a}}$	$20.16\pm 0.19^{\text{b}}$	$18.09\pm 0.27^{\text{c}}$	$18.44\pm 0.19^{\text{c}}$	$19.48\pm 0.23^{\text{d}}$	$15.00\pm 0.29^{\text{e}}$
CC	$93.80\pm 0.63^{\text{a}}$	$95.02\pm 0.55^{\text{a}}$	$91.09\pm 0.76^{\text{b}}$	$100.13\pm 0.53^{\text{c}}$	$100.27\pm 0.66^{\text{c}}$	$81.86\pm 0.82^{\text{d}}$
CBC	$7.85\pm 0.06^{\text{a}}$	$7.63\pm 0.05^{\text{b}}$	$8.35\pm 0.07^{\text{c}}$	$8.91\pm 0.05^{\text{d}}$	$8.95\pm 0.06^{\text{d}}$	$7.61\pm 0.07^{\text{b}}$
BW	$61.29\pm 0.90^{\text{a}}$	$54.93\pm 0.78^{\text{b}}$	$54.12\pm 1.09^{\text{b}}$	$72.10\pm 0.76^{\text{c}}$	$76.36\pm 0.94^{\text{d}}$	$37.11\pm 1.17^{\text{e}}$

WH – wither height, RH – rump height, BL – body length, CD – chest depth, CW – chest width, RW – rump width, CC – chest circumference, CBC – cannon bone circumference, BW – body weight. Different letters in the superscript indicate a statistically significant difference between means (P<0.05) in the same row.

## 3 Results

Least square means (LSMs) and standard errors (SEs) for morphometric measures and body weight of six sheep breeds are presented in Table 1. The highest wither height was found in the Montenegrin breeds Sjenička and Pivska Pramenka, followed by Slovenian Istrian Pramenka (72.71, 71.33, and 69.62 cm), with significant differences (P<0.05) among all studied breeds, while Bela Krajina (65.56 cm) was not significantly different from Bardoka and Zeta Žuja (66.22 and 64.76 cm). The breeds were in the same order regarding the average values of other morphometric traits like rump height, chest circumference, and body weight. The average body length, ranging from 59.41 cm (Zeta Žuja) to 79.39 cm (Istrian Pramenka), is the only morphometric measure that was significantly different (P<0.05) among all breeds.

**Table 2:** The least square means (LSMs) with standard errors (SEs) of morphometric indices for six sheep breeds.

	Sheep breeds					
Indices	Istrian	Bela Krajina	Bardoka	Pivska	Sjenička	Zeta Žuja
	Pramenka	Pramenka		Pramenka	sheep	Žuja
IBF	$114.13\pm 0.56^{\text{a}}$	$110.89\pm 0.56^{\text{b}}$	$99.95\pm 0.55^{\text{c}}$	$99.29\pm 0.42^{\text{c}}$	$107.15\pm 0.43^{\text{d}}$	$91.80\pm 0.69^{\text{e}}$
CI	$67.67\pm 0.92^{\text{a}}$	$68.58\pm 0.55^{\text{a}}$	$64.92\pm 1.01^{\text{b}}$	$67.92\pm 0.58^{\text{a}}$	$64.62\pm 0.79^{\text{b}}$	$55.55\pm 0.70^{\text{c}}$
IH	$101.57\pm 0.30^{\text{a}}$	$100.31\pm 0.26^{\text{b}}$	$99.89\pm 0.22^{\text{b}}$	$99.20\pm 0.26^{\text{b}}$	$99.43\pm 0.14^{\text{b}}$	$99.58\pm 0.24^{\text{b}}$
CDI	$48.26\pm 0.39^{\text{a}}$	$49.89\pm 0.25^{\text{b}}$	$43.97\pm 0.30^{\text{c}}$	$45.03\pm 0.21^{\text{d}}$	$45.40\pm 0.94^{\text{d}}$	$42.11\pm 0.32^{\text{e}}$
ITD	$134.77\pm 1.10^{\text{a}}$	$145.07\pm 0.89^{\text{b}}$	$137.66\pm 0.96^{\text{c}}$	$140.51\pm 0.67^{\text{d}}$	$137.92\pm 0.93^{\text{c}}$	$126.50\pm 1.06^{\text{d}}$
DTI	$8.40\pm 0.07^{\text{a}}$	$8.05\pm 0.05^{\text{b}}$	$9.18\pm 0.08^{\text{c}}$	$8.91\pm 0.05^{\text{d}}$	$8.94\pm 0.07^{\text{d}}$	$9.31\pm 0.10^{\text{c}}$
BCI	$126.93\pm 2.13^{\text{a}}$	$138.17\pm 1.58^{\text{b}}$	$125.57\pm 1.53^{\text{a}}$	$140.86\pm 1.11^{\text{b}}$	$138.64\pm 1.89^{\text{b}}$	$103.74\pm 1.61^{\text{c}}$
RCBI	$12.62\pm 0.12^{\text{a}}$	$11.64\pm 0.09^{\text{b}}$	$11.28\pm 0.10^{\text{c}}$	$12.50\pm 0.11^{\text{a}}$	$12.31\pm 0.10^{\text{d}}$	$11.75\pm 0.12^{\text{b}}$
IBW	$87.95\pm 1.45^{\text{a}}$	$83.79\pm 1.03^{\text{a}}$	$81.72\pm 1.01^{\text{b}}$	$101.09\pm 0.90^{\text{c}}$	$104.92\pm 1.58^{\text{d}}$	$57.20\pm 1.06^{\text{e}}$

IBF – index of body frame, CI – chest index, IH – index of height, CDI – chest depth index, ITD – index of thorax development, DTI – dactyl thorax index, BCI – Baron–Crevat index, RCBI – relative cannon bone index, IBW – index of body weight. Different letters in superscript indicate a statistically significant difference between means (P<0.05) in the same row.

The morphometric indices were used to describe the proportions among the body parts of animals. The obtained values of body frame index (IBF) showed that Slovenian Bela Krajina, Istrian Pramenka, and Montenegrin Sjenička breeds (114, 111, and 107, respectively) have rectangular body frames (Table 2), Bardoka and Pivska Pramenka have square body frames (99.3 and 100), and Zeta Žuja has a short body frame (IBF = 91.8). The chest index (CI) of all studied breeds ranged from 64.6 to 68.6 and showed good skeletal fitness of all breeds except Zeta Žuja, for which CI was significantly lower (55.56) compared to other breeds (P<0.01).

The obtained values of ITD and CDI, as very important parameters for the estimation of body conformation, indicated very good development of the thorax in all breeds. That was especially expressed in Bela Krajina and Pivska Pramenka breeds, both with ITD above 140, while the value of CDI was above 45 for all breeds except Bardoka and Zeta Žuja. The values of BCI indicated that Pivska Pramenka is one of the strongest breeds, with an average index 140.86, followed by Sjenička sheep and Bela Krajina Pramenka (138.6 and 138.2). Both Slovenian breeds had lower DTI values (8.05 and 8.40) than Montenegrin breeds, ranging from 8.91 (Pivska Pramenka) to 9.31 (Zeta Žuja), with significant differences among breeds (P<0.05).

**Table 3:** Pearson's correlation coefficients between the morphometric traits of investigated sheep breeds.

	WH	RH	BL	CD	CW	RW	CC	CBC	BW
WH	1.00								
RH	0.92${}^{*}$	1.00							
BL	0.52${}^{*}$	0.45${}^{*}$	1.00						
CD	0.50${}^{*}$	0.44${}^{*}$	0.74${}^{*}$	1.00					
CW	0.31${}^{*}$	0.28${}^{*}$	0.63${}^{*}$	0.68${}^{*}$	1.00				
RW	0.22${}^{*}$	0.16${}^{*}$	0.63${}^{*}$	0.63${}^{*}$	0.69${}^{*}$	1.00			
CC	0.58${}^{*}$	0.60${}^{*}$	0.51${}^{*}$	0.69${}^{*}$	0.65${}^{*}$	0.43${}^{*}$	1.00		
CBC	0.66${}^{*}$	0.72${}^{*}$	0.24${}^{*}$	0.27${}^{*}$	0.19${}^{*}$	0.02${}^{\text{ns}}$	0.59${}^{*}$	1.00	
BW	0.75${}^{*}$	0.77${}^{*}$	0.61${}^{*}$	0.65${}^{*}$	0.56${}^{*}$	0.36${}^{*}$	0.87${}^{*}$	0.75${}^{*}$	1.00

WH – wither height, RH – rump height, BL – body length, CD – chest depth, CW – chest width, RW – rump width, CC – chest circumference, CBC – cannon bone circumference, BW – body weight. ${}^{*}$ Statistically significant correlation (P<0.001), ns – non-significant correlation (P>0.05).

The total phenotypic correlations among all morphometric measures for all animals and studied breeds are presented in Table 3. Most of these correlations were moderately positive and statistically significant (P<0.001), except the correlation between CBC and RW, which was very low and not significant. The strongest correlation was between WH and RH (0.92) and between BW and WH, RH, CC, and CBC (0.75, 0.77, 0.87, and 0.75, respectively). Relatively low correlations (≤0.3) were among RW, WH, and RH (0.22 and 0.16), as well as correlations of CBC with BL, CD, CW, and RW (0.24, 0.27, 0.19, and 0.02). The correlation coefficients observed within each of the included breeds (Tables S3a, b, c in the Supplement) had lower values in general, but with similar trends as the total correlation coefficients for all breeds, as shown above. All Montenegrin sheep breeds were particularly characterized by a very low correlation of RW and CC with the measures of body height, length (WH, RH, BL), and even BW, which were not significant (P>0.05) and in some cases were even negative.

**Table 4:** Eigenvalues, total and accumulated variance, and factor and factor loadings for the morphometric parameters (measures and indices) of six sheep breeds.

Type traits and indices	Factor 1	Factor 2	Factor 3	Communality
WH	0.104	**0.988**	0.110	0.999
RH	-0.052	**0.984**	0.157	0.995
BL	**0.736**	0.610	0.221	0.963
CD	**0.732**	0.561	0.369	0.986
CW	**0.710**	0.452	0.530	0.988
RW	**0.841**	0.332	0.386	0.967
CC	0.259	**0.729**	0.632	0.999
CBC	-0.386	**0.831**	0.362	0.971
BW	0.174	**0.875**	0.447	0.996
IBF	**0.918**	0.205	0.258	0.951
CI	0.598	0.304	0.683	0.917
IH	**0.889**	-0.201	-0.282	0.910
CDI	**0.888**	-0.039	0.431	0.976
ITD	0.333	0.112	**0.935**	0.997
DTI	**-0.876**	0.121	-0.386	0.930
BCI	0.304	0.526	**0.792**	0.996
RCBI	0.389	**0.801**	-0.263	0.861
IBW	0.210	**0.813**	0.536	0.993
Eigenvalue	11 404	4.340	1.6543	
Total variance (%)	63 356	24 108	9.190	
Cumulative variance ( %)	**63 356**	**87 464**	**96 654**	

WH – wither height, RH – rump height, BL – body length, CD – chest depth, CW – chest width, RW – rump width, CC – chest circumference, CBC – cannon bone circumference, BW – body weight, IBF – index of body frame, CI – chest index, IH – index of height, CDI – chest depth index, ITD – index of thorax development, DTI – dactyl thorax index, BCI – Baron–Crevat index, RCBI – relative cannon bone index, IBW – index of body weight. Marked loading was >0.70; higher values are bold.

Principal component analysis (PCA) based on the average values of all morphometric parameters for six studied sheep breeds was performed after varimax normalized rotation (Table 4). The presented coefficients show the relative contribution of each measurement to a particular principal component (factor), whilst the percentage of total variance was used as a basis to determine how the total component solutions account for the variables (measurements) represented. The analyses showed that the first two factors contributed 87.5 % of the total variance, while the first three factors accounted for 96.6 % of the total accumulated variance, which satisfactorily explained the differences expressed in the evaluated traits.

The first principal component with an eigenvalue of 11.4 accounted for 63.4 % of the total variance, and it is strongly correlated with four body measures (BL, CD, CW, and RW) and three morphometric indices (IBF, IH, and CDI). All these parameters had a very high positive contribution to the variation and breed differentiation, which suggests they are correlated. Only DTI had a high negative contribution for the first principal component. The second factor had an eigenvalue of 4.3 and accounted for 24 % of the total variance, which was associated with a very high and positive contribution of WH and RH, as well as CC, CBC, BW, and the indices RCBI and IBW.

**Figure 2:**
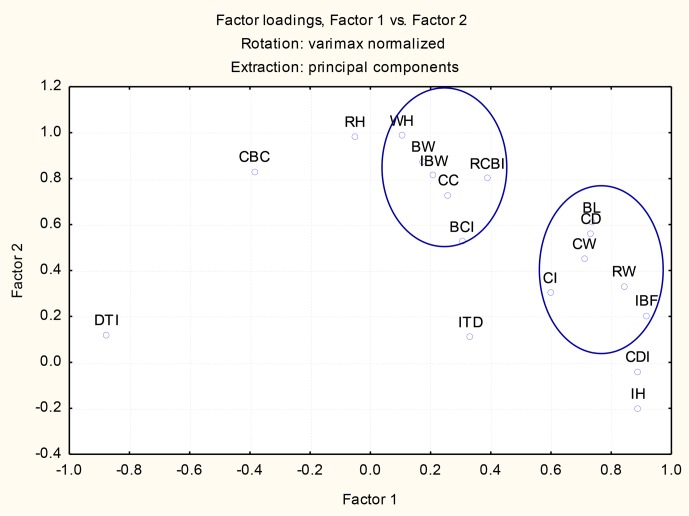
Scatter plot of the principal component analysis (Factor 1 vs. Factor 2).

After 2-D varimax transformation of the first two factors, which accounted for more than 80 % of the total variance, most of the morphometric measures were clustered into two groups (Fig. 2). One was formed by BL, CD, CW, and RW with related indices, while the second group was formed by WH, RH, BW, CC, and CBC. Three negative variables for Factor 1 were identified (DTI, CBC, and CH), for which DTI had a very high negative contribution, while in Factor 2, the negative variables CDI and IH were identified, both with a relatively low contribution.

**Table 5:** Euclidean distances among six sheep breeds based on morphological measurements and indices.

	Istrian	Bela	Bardoka	Pivska	Sjenička	Zeta
	Pramenka	Krajina		Pramenka		Žuja
Istrska Pramenka	0.00					
Bela Krajina	3.72	0.00				
Bardoka	5.58	4.70	0.00			
Pivska Pramenka	4.97	5.27	4.63	0.00		
Sjenička	4.54	5.50	5.20	1.87	0.00	
Zeta Žuja	8.67	8.38	4.89	8.34	8.87	0.00

**Figure 3:**
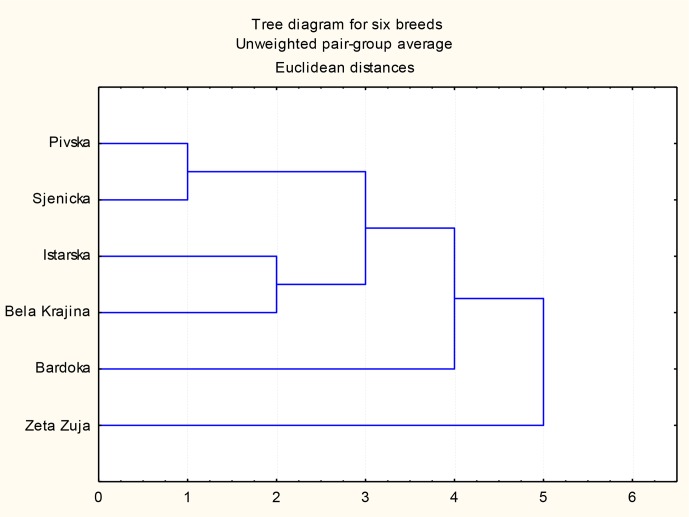
UPGMA tree diagram of distances among six sheep breeds obtained based on means for morphometric parameters.

Based on the average data for nine morphometric measures and nine body indices of six breeds, UPGMA analyses were performed and a tree diagram (Fig. 3) was constructed, showing the variability and distances among the included breeds clustered in groups. The values of the most convenient Euclidean distances among breeds are presented in Table 5. The largest distance was between Zeta Žuja and other investigated sheep breeds for which distances ranged from 8 to 9, except Bardoka. Lower distances were between Sjenička and Pivska Pramenka (only 1.9) and between Istrian and Bela Krajina Pramenka (3.7). The Bardoka breed has a fairly equal distance to the other five breeds that ranged from 4.6 (Pivska Pramenka) to 5.6 (Istrian Pramenka).

## 4 Discussion

Regarding the morphometric measures, many differences among Montenegrin and Slovenian sheep breeds were identified. Among all six sheep breeds Sjenička had the largest body weight and some body measurements (WH, RH, CC, and CBC), followed by Pivska, Istrian, and Bela Krajina Pramenka, then the Bardoka and Zeta Žuja breeds, which were the lowest among all included breeds. The Slovenian sheep breeds (Istrian and Bela Krajina Pramenka) had a larger chest depth and chest width than Montenegrin sheep breeds. Istrian Pramenka had the largest body length among all included breeds, although the body weight was significantly lower than the Montenegrin Sjenička breed by 19.7 % and Pivska Pramenka by 15 %. The rump height of all Montenegrin sheep breeds (Bardoka, Pivska Pramenka, Sjenička sheep, and Zeta Žuja) was slightly higher than wither height, while in both Slovenian sheep breeds the wither was higher than the rump, which is similar to the results obtained by Salako (2006) and Yunsa et al. (2013).

Morphometric indices were calculated from morphometric measures of body size and are generally used for the estimation of proportions and conformation of animals (Pares-Casanova et al., 2013). Based on the value of IBF, the Bela Krajina, Istrian, and Sjenička breeds have a rectangular body frame, while Bardoka and Pivska Pramenka are square. These results are in accordance with values of IBF reported by Handiwiravan et al. (2011) for four local sheep breeds.

ITD is important for the animals in terms of their fitness, good thorax development, and capacity of the respiratory system, especially for breeds reared at higher altitudes (Esquivelzeta et al., 2011; Chacon et al., 2011), as is the case with most Montenegrin and Slovenian sheep breeds.

The dactyl thorax index (DTI) provides information about conformation and whether an animal is more appropriate for meat or milk production (Bravo and Supelveda, 2010; Cerqueira et al., 2011; Khargharai et al., 2015). Relatively low values of DTI (lower than 10) were obtained for all investigated breeds, which means these breeds consist of relatively light animals that are very well adapted to walking longer distances and more appropriate for milk production than for meat production, especially Istrian, Bela Krajina, and Bardoka.

The body frame (type, structure, and/or proportions) should be correlated with the breed purpose. Theoretically, the type and performance (function) are in low genetic correlation and they are likely to be independently inherited (Salako, 2006). Diversity among breeds is classically considered a major criterion to be taken into account when setting priorities for the conservation of domestic animal breeds (Yakubu and Ibrahim, 2011).

The DTI indicates the conformation and the body frame of an animal, allowing for the establishment of a relationship between the body weight of an individual and the morphometric measures that support it. Likewise, it gives information about the degree of fineness of the skeleton, which has the highest value in fattened animals.

The applied linear model showed that the effect of the breed significantly affected all morphometric parameters, with the coefficient of determination ($R^{2}$) above 0.5 for all body measures, except RW and the indices CI and IH. The morphological differences among breeds are an indication of the inherent genetic constitution of each sheep breed.

The strong correlation between BW and some morphometric measures (WH, RW, CC, and CBC) means that these measures could be used for the estimation of body weight. Similar to this study, high correlations among the mentioned measures were found by Salako (2006) and Mavule et al. (2013). On the other hand, the low correlation between CBC and BL, CD, CW, and RW is in line with results reported by Popoola (2015) and Cilek and Petkova (2016).

Since PCA was used as a method for reducing the number of variables, the value of the CI variable (lower than 0.7 for all three factors) could be excluded from the further morphometric characterization of sheep breeds. The morphometric traits in the same component were classified together, and it may be concluded that they probably have common genomic positions for their genetic control (Khargharai et al., 2015). The accumulation of most of the variance in the first principal component is, according to Yakubu et al. (2011), characteristic of animals with larger body measures. The proportion of variance accounted for in the original variables is very high, from 0.861 to 0.999. The relevance of the PCA is evident from the reduction of a larger number of variables into components that gave a better description of body frame and body conformation.

According to the morphometric parameters, the UPGMA tree diagram shows two groups or clusters. In the first group, Montenegrin Pivska Pramenka and Sjenička sheep breeds were clustered together with a relatively short distance. Slovenian Istrian and Bela Krajina Pramenka breeds made their own cluster with a medium distance, while the other two Montenegrin breeds (Bardoka and Zeta Žuja) were grouped separately with a relatively long distance, the especially Zeta Žuja breed.

Generally, such phenotypic divergence among breeds might be partly associated with differences in the production systems, agro-climatic conditions, and natural resources (Yadav et al., 2013). The breeding objectives depending on the breed purpose could be an important effect as well. The studied breeds are reared in geographically different areas, some of them in rather isolated areas, without frequent mixing of the breeding animals, which also contributes to the phenotypic divergences among them. Thus, larger differences in body measures imply larger distances among breeds. The clustering of the Slovenian sheep breeds (Istrian and Bela Krajina Pramenka) in one independent cluster was most probably affected by the quite large geographical distance between Slovenia and Montenegro, as well as larger differences in rearing conditions between two countries.

## 5 Conclusions

Different sheep breeds, especially local ones, are sometimes the only farm animal resource in the specific agro-climatic conditions of less favoured mountain areas. Together with other ruminant species, they are fundamental for the maintenance of social and economic stability in rural areas.

The morphometric characterization of Montenegrin and Slovenian sheep breeds, reared mostly in extensive production systems, would enable an accurate comparison of them with other breeds. The differentiation between Montenegrin and Slovenian breeds was made based on morphometric traits (body measures, body weight, and indices) using different procedures.

The results of this study could essentially contribute to the characterization of the studied breeds and could provide basic information to assist in conservation and breeding programme creation. Variation and diversity in the morphological traits of the sheep breeds identified in this study should be further confirmed by research on genetic markers in the future.

## Supplement

10.5194/aab-62-393-2019-supplement The supplement related to this article is available online at: https://doi.org/10.5194/aab-62-393-2019-supplement.

## Data Availability

The data are available from the corresponding author upon request.
