# Supplementary material for: Differentiation of some Pramenka sheep breeds based on morphometric characteristics
Source: Arch Anim Breed. 2019 Jul 10;62(2):393–402. doi: 10.5194/aab-62-393-2019 (PMC6859914; doi:10.5194/aab-62-393-2019)
Supplement: The supplement related to this article is available online at: https://doi.org/10.5194/aab-62-393-2019-supplement. [file aab-62-393-supplement.pdf]

Supplement of Arch. Anim. Breed., 62, 393–402, 2019
https://doi.org/10.5194/aab-62-393-2019-supplement
© Author(s) 2019. This work is distributed under
the Creative Commons Attribution 4.0 License.

Open Access
Archives Animal Breeding

Supplement of

Differentiation of some Pramenka sheep breeds based on morphometric characteristics

Božidarka Marković et al.

Correspondence to: Božidarka Marković (bozidarkam@ucg.ac.me)

The copyright of individual parts of the supplement might differ from the CC BY 4.0 License.

Supplement Tables:

Table 3a: Pearson correlation coefficients of morphometric traits of Istrian Pramenka (below diagonal) and Bela Krajina Pramenka (above diagonal)

**Pearson Correlation Coefficients, I = 64 BK 64**<br>**Prob > |r| under H0: Rho=0**									
	**WH**	**RH**	**BL**	**CD**	**CW**	**RW**	**CC**	**CBC**	**BW**
**WH**	1	0,804<br><.0001	0,382<br>0,000	0,452<br><.0001	0,280<br>0,009	0,279<br>0,009	0,352<br>0,001	0,376<br>0,000	0,345<br>0,001
**RH**	0,791<br><.0001	1	0,385<br>0,000	0,502<br><.0001	0,319<br>0,003	0,290<br>0,007	0,412<br><.0001	0,445<br><.0001	0,481<br><.0001
**BL**	0,425<br>0,001	0,345<br>0,005	1	0,462<br><.0001	0,364<br>0,001	0,263<br>0,014	0,398<br>0,000	0,486<br><.0001	0,507<br><.0001
**CD**	0,402<br>0,001	0,486<br><.0001	0,338<br>0,006	1	0,580<br><.0001	0,406<br>0,000	0,687<br><.0001	0,486<br><.0001	0,769<br><.0001
**CW**	0,177<br>0,161	0,239<br>0,058	0,220<br>0,080	0,412<br>0,001	1	0,523<br><.0001	0,714<br><.0001	0,579<br><.0001	0,731<br><.0001
**RW**	0,375<br>0,002	0,434<br>0,000	0,432<br>0,000	0,449<br>0,000	0,547<br><.0001	1	0,342<br>0,001	0,416<br><.0001	0,466<br><.0001
**CC**	0,451<br>0,000	0,522<br><.0001	0,277<br>0,027	0,685<br><.0001	0,575<br><.0001	0,658<br><.0001	1	0,464<br><.0001	0,790<br><.0001
**CBC**	0,210<br>0,096	0,292<br>0,019	0,355<br>0,004	0,536<br><.0001	0,470<br><.0001	0,522<br><.0001	0,542<br><.0001	1	0,555<br><.0001
**BW**	0,438<br>0,000	0,491<br><.0001	0,418<br>0,001	0,737<br><.0001	0,667<br><.0001	0,695<br><.0001	0,839<br><.0001	0,745<br><.0001	1

WH – wither height, RH – rump height, BL – body length, CD – chest depth, CW – chest width, RW – rump width, CC – chest circumference, CBC – cannon bone circumference, BW – body weight.

Table 3b: Pearson correlation coefficients of morphometric traits of Pivska Pramenka (below diagonal) and Sjenicka sheep (above diagonal)

Pearson Correlation Coefficients, P = 90 S = 59
Prob > |r| under H0: Rho=0

	WH	RH	BL	CD	CW	RW	CC	CBC	BW
**WH**	1	0,926<br><.0001	0,516<br><.0001	0,494<br><.0001	0,203<br>0,122	0,180<br>0,172	0,406<br>0,001	0,497<br><.0001	0,491<br><.0001
**RH**	0,667<br><.0001	1	0,549<br><.0001	0,424<br>0,001	0,269<br>0,039	0,225<br>0,086	0,329<br>0,011	0,399<br>0,002	0,435<br>0,001
**BL**	0,331<br>0,001	0,475<br><.0001	1	0,380<br>0,003	0,461<br>0,000	0,403<br>0,002	0,333<br>0,010	0,398<br>0,002	0,556<br><.0001
**CD**	0,189<br>0,074	0,117<br>0,273	0,016<br>0,882	1	0,405<br>0,002	0,509<br><.0001	0,814<br><.0001	0,594<br><.0001	0,818<br><.0001
**CW**	0,014<br>0,894	0,135<br>0,205	0,182<br>0,086	0,186<br>0,079	1	0,741<br><.0001	0,523<br><.0001	0,108<br>0,414	0,583<br><.0001
**RW**	-0,082<br>0,440	0,029<br>0,790	0,066<br>0,534	0,224<br>0,034	0,620<br><.0001	1	0,559<br><.0001	0,191<br>0,146	0,602<br><.0001
**CC**	-0,048<br>0,655	-0,082<br>0,443	0,014<br>0,895	0,081<br>0,450	0,276<br>0,009	-0,014<br>0,896	1	0,491<br><.0001	0,968<br><.0001
**CBC**	0,344<br>0,001	0,321<br>0,002	0,371<br>0,000	-0,156<br>0,143	0,001<br>0,995	-0,171<br>0,108	0,216<br>0,041	1	0,530<br><.0001
**BW**	0,305<br>0,004	0,338<br>0,001	0,460<br><.0001	0,056<br>0,600	0,108<br>0,310	-0,339<br>0,001	0,454<br><.0001	0,457<br><.0001	1

WH – wither height, RH – rump height, BL – body length, CD – chest depth, CW – chest width, RW – rump width, CC – chest circumference, CBC – cannon bone circumference, BW – body weight.

Table 3c: Pearson correlation coefficients of morphometric traits of Bardoka (below diagonal) and Žuja (above diagonal)

Pearson Correlation Coefficients, B = 44, Z=38
Prob > |r| under H0: Rho=0

	WH	RH	BL	CD	CW	RW	CC	CBC	BW
**WH**	1	0,936	0,538	0,648	0,444	0,147	0,433	0,403	0,627
		<.0001	0,001	<.0001	0,005	0,378	0,007	0,012	<.0001
**RH**	0,895	1	0,578	0,529	0,363	0,050	0,508	0,404	0,672
	<.0001		0,000	0,001	0,025	0,764	0,001	0,012	<.0001
**BL**	0,402	0,359	1	0,710	0,495	0,029	0,498	0,656	0,768
	0,007	0,017		<.0001	0,002	0,861	0,002	<.0001	<.0001
**CD**	0,594	0,755	0,217	1	0,499	0,234	0,547	0,622	0,731
	<.0001	<.0001	0,157		0,001	0,157	0,000	<.0001	<.0001
**CW**	-0,263	-0,206	-0,196	-0,160	1	-0,037	0,512	0,583	0,632
	0,084	0,180	0,203	0,299		0,827	0,001	0,000	<.0001
**RW**	0,159	0,178	0,031	0,112	0,357	1	0,010	0,108	-0,048
	0,304	0,248	0,844	0,468	0,017		0,954	0,518	0,774
**CC**	0,248	0,264	-0,006	0,448	0,316	0,246	1	0,463	0,740
	0,105	0,084	0,972	0,002	0,037	0,108		0,003	<.0001
**CBC**	0,505	0,545	0,389	0,469	-0,171	-0,049	0,323	1	0,714
	0,001	0,000	0,009	0,001	0,268	0,753	0,032		<.0001
**BW**	0,388	0,387	0,398	0,502	0,209	0,239	0,914	0,450	1
	0,009	0,010	0,008	0,001	0,174	0,118	<.0001	0,002	

WH – wither height, RH – rump height, BL – body length, CD – chest depth, CW – chest width, RW – rump width, CC – chest circumference, CBC – cannon bone circumference, BW – body weight.
